# Supplementary material for: Streptococcus australis and Ralstonia pickettii as Major Microbiota in Mesotheliomas
Source: J Pers Med. 2021 Apr 14;11(4):297. doi: 10.3390/jpm11040297 (PMC8070724; doi:10.3390/jpm11040297)
Supplement: Supplementary file 1 [file jpm-11-00297-s001.pdf]

## **Supplementary Figure S1.**

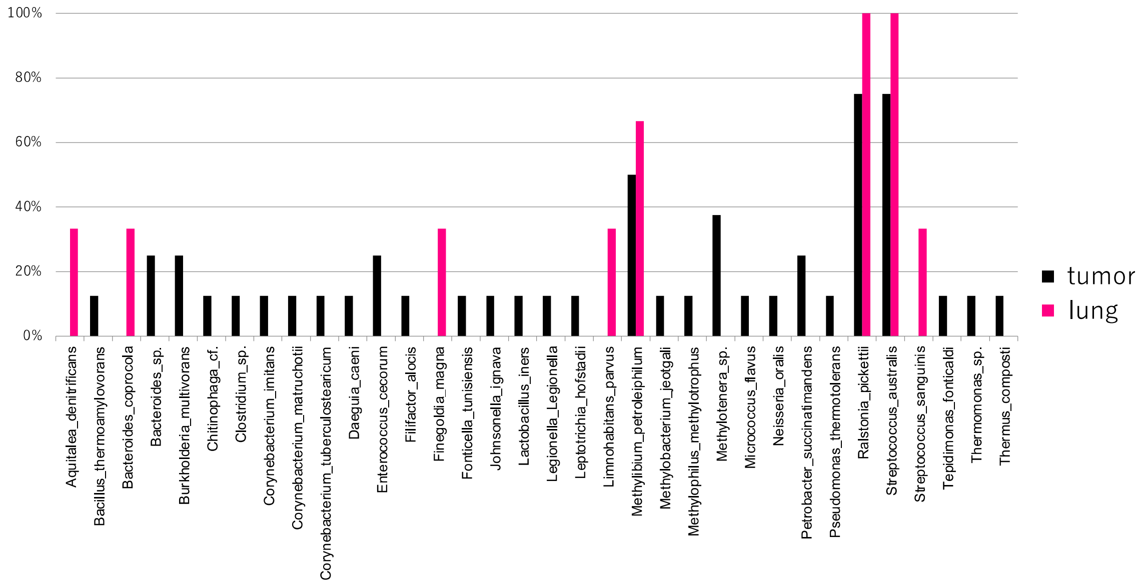


Composition of detected species in all samples. Composition of *Streptococcus australis* and *Ralstonia pickettii* was higher both in tumor (as shown in black bar) and lung (as shown in red bar) samples.
